# Case Report: A rare co-occurrence of IgA pemphigus and pyoderma gangrenosum associated with IgA-κ type monoclonal gammopathy of undetermined significance: a 19-year diagnostic and therapeutic journey

**DOI:** 10.3389/fimmu.2026.1832860

**Published:** 2026-05-20

**Authors:** You Xie, Xinqiao Liu, Juan Wu, Liuqing Chen, Jinbo Chen

**Affiliations:** 1 Department of Dermatology, Traditional Chinese and Western Medicine Hospital of Wuhan, Tongji Medical College, Huazhong University of Science and Technology, Wuhan, Hubei, China; 2 Hubei Province & Key Laboratory of Skin Infection And Immunity, Wuhan, Hubei, China

**Keywords:** dapsone, IgA pemphigus, monoclonal gammopathy, neutrophilic dermatosis, pyoderma gangrenosum

## Abstract

We report a complex case of neutrophilic dermatosis (ND) in a 61-year-old woman with a 19-year history of recurrent, pruritic, and painful vesiculopustular eruptions. Her clinical course was marked by evolving diagnoses, from pustular vasculitis to IgA pemphigus and pyoderma gangrenosum (PG). Concurrent investigations revealed an IgA-κ type monoclonal gammopathy of undetermined significance (MGUS). This case highlights the rare association between neutrophilic dermatoses (ND) and haematological disorders, and illustrates the therapeutic challenges posed by comorbidities such as osteoporosis and a history of tuberculosis. Management with dapsone and low-dose corticosteroids led to significant clinical improvement.

## Introduction

Neutrophilic dermatoses (NDs) represent a spectrum of disorders characterized by sterile, neutrophil-rich cutaneous infiltrates, often associated with systemic diseases (1). IgA pemphigus and pyoderma gangrenosum (PG) are distinct entities within this spectrum. Their coexistence in a single patient is exceedingly rare. Furthermore, both conditions are known to have associations with haematological dyscrasias, particularly monoclonal gammopathy of undetermined significance (MGUS) (2, 3). We present a detailed longitudinal case illustrating the diagnostic evolution and therapeutic challenges in managing concurrent IgA pemphigus and PG associated with IgA-κ MGUS.

## Case presentation

A 61-year-old woman presented with an 18-year history of recurrent blisters and sterile pustules. The initial lesions appeared on her hands and feet in 2005, progressing to pustules. Symptoms were partially controlled with over-the-counter topical corticosteroids (“Skin Expert Ointment”).

**Figure 1:**
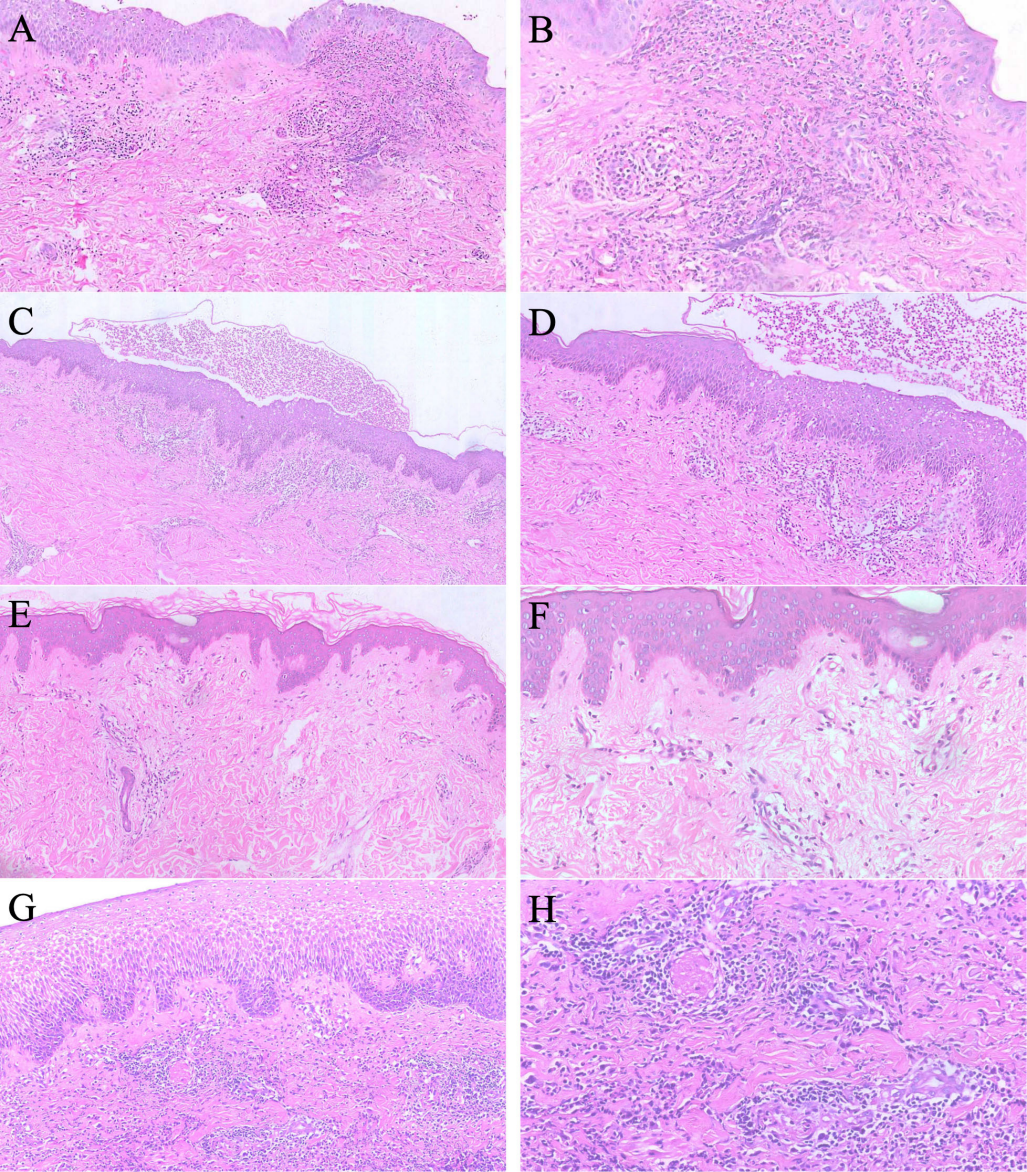
Previous biopsy results from our hospital prior to admission were as follows: **(A, B)** A skin biopsy of the external genitalia performed on November 19, 2014, revealed epidermal hyperplasia and intercellular edema. The dermis showed infiltration of lymphocytes, neutrophils, and histiocytes, with vascular wall destruction and fibrinoid deposition. Perivascular inflammatory cell infiltration was also observed. The findings are largely consistent with changes of pustular vasculitis. **(C, D)** a skin biopsy of the back performed on December 3, 2018, revealed subcorneal pustule formation and mild epidermal hyperplasia. Neutrophil migration was observed within the epidermis. The superficial to mid-dermis showed numerous neutrophils, variable lymphocytes, and abundant plasma cell infiltration. On one side of the section, numerous extravascular red blood cells were noted, surrounded by neutrophils and nuclear dust. The findings were consistent with changes of neutrophilic dermatosis. **(E, F)** A skin biopsy of the back performed on April 12, 2019, revealed no subepidermal pustules. Perivascular lymphohistiocytic infiltration was observed in the superficial dermis, with a few eosinophils and neutrophils present. The findings do not rule out neutrophilic dermatosis, and systemic evaluation should be recommended. **(G, H)** A skin biopsy of the left breast performed on April 14, 2023, revealed epidermal hyperplasia and intercellular edema. The dermis showed infiltration of lymphocytes, neutrophils, and histiocytes, with vascular wall swelling and thrombus formation. The findings were consistent with changes of vasculitis.

In 2009, lesions extended to her trunk and limbs. She received a course of systemic antibiotics and corticosteroids (details unknown) for presumed “eczema with secondary infection” with temporary improvement. In 2014, she developed vulvar vesiculopustular lesions that ulcerated. A skin biopsy in November 2014 showed vascular wall swelling and a dermal infiltrate of lymphocytes, neutrophils, and histiocytes, leading to a diagnosis of pustular vasculitis (**Figures 1A, B**). Treatment with oral Tripterygium Glycosides Tablets and topical steroids/antibiotics was effective but self-discontinued.

In October 2018, a severe recurrence on her back prompted re-hospitalisation. A repeat biopsy again suggested pustular vasculitis (**Figures 1C, D**). Treatment with colchicine (1 mg/day) and thalidomide (50 mg/day) controlled lesions for one month, but they recurred upon cessation.

In April 2019, a further exacerbation led to another biopsy showing a perivascular histiocytic infiltrate with eosinophils and neutrophils (**Figures 1E, F**). Direct immunofluorescence and acid-fast staining were negative. The working diagnosis remained pustular vasculitis, and she responded to oral acitretin (20 mg/day) until self-discontinuation.

In June 2022, she presented with worsening vulvar lesions. Treatment with acitretin and colchicine, later switched to acitretin and cyclosporine, was halted after two months due to chest pain; a CT scan showed punctate lung opacities.

In March 2023, a new ulcerative lesion developed on her left breast. A biopsy in April 2023 demonstrated dermal infiltration of lymphocytes, neutrophils, and histiocytes with thrombosis, consistent with PG (**Figures 1G, H**). She received a short course of cyclosporine but self-discontinued. In May 2023, she was hospitalised for PG and treated with intravenous dexamethasone (tapering regimen) and oral total glucosides of paeony, with good response. During outpatient tapering of oral methylprednisolone to 4 mg/day (August 2024), a severe flare with new lesions prompted re-admission.

### Past medical history

Notable for pulmonary, cutaneous, and lymphatic tuberculosis (treated in 2014), hypertension, coronary heart disease, osteoporosis (with a compression fracture), and post-cholecystectomy status (2005).

### Physical examination (cutaneous)

**Figure 2:**
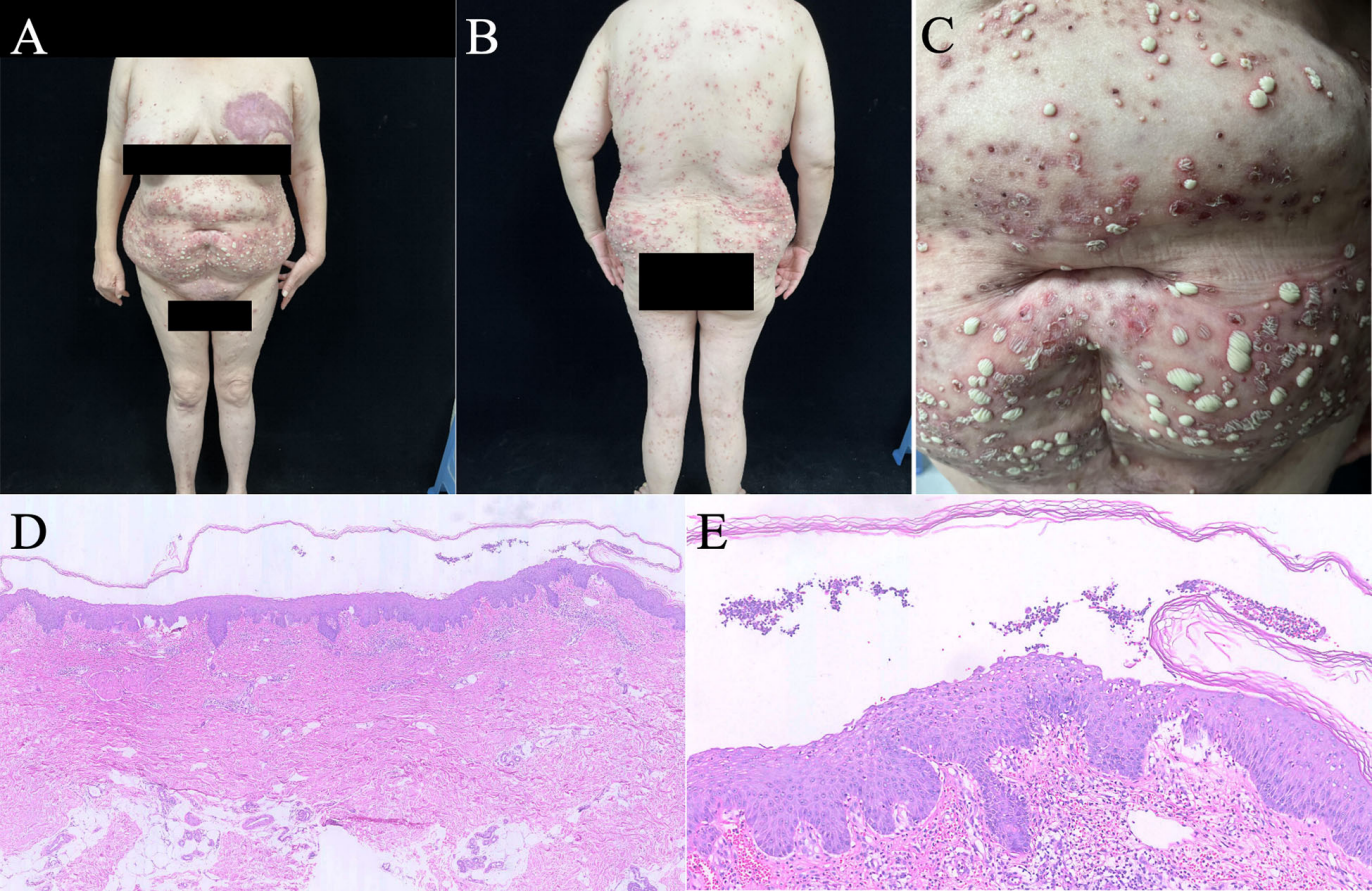
Clinical photographs on admission showing widespread vesiculopustular lesions and ulcers on the trunk and limbs, close-up of pustules and erosions, cribriform scarring on the left breast and the vulva **(A–C)**. Histopathology from the left thigh biopsy revealed subcorneal pustule formation with the presence of acantholytic cells, as well as epidermal hyperplasia and intraepidermal neutrophil migration **(D, E)**.

Multiple vesicles, erosions, ulcers, and pustules (millet to broad bean-sized) were observed on the trunk, limbs, and vulva, with a proximal predominance. Some pustules were surrounded by an erythematous halo or coalesced into “pus lakes.” A positive Nikolsky sign was noted on some bullae. Old cribriform scars were present on the vulva and left breast (**Figures 2A–C**).

### Investigations and diagnosis

*Histopathology (left thigh):* revealed subcorneal pustules, intraepidermal neutrophils, acantholytic keratinocytes, and a perivascular neutrophilic/lymphocytic infiltrate in the superficial to mid-dermis (**Figures 2D, E**).

*Immunopathology:* direct and indirect immunofluorescence were negative for IgA, IgG, IgM, and C3. Serum antibodies against BP180, BP230, DSG1, and DSG3 were also negative.

*Working diagnosis:* based on the clinical phenotype and histopathological finding of acantholysis, a diagnosis of IgA pemphigus was made despite negative immunofluorescence.

*Haematological work-up:* serial immunofixation electrophoresis (2018, 2019, 2023) consistently showed an abnormal IgA-κ type M-band. Urinary free κ light chains and κ/λ ratios progressively increased. A bone marrow biopsy showed active proliferation with easily visible plasma cells, confirming IgA-type MGUS.

*Exclusion of other conditions:* comprehensive serology (ANA, ANCA, immunoglobulins, RF), infectious screens (hepatitis viruses, HIV, syphilis, CMV, EBV), and imaging (chest/abdominal CT) ruled out active infection, solid malignancy, or other definitive autoimmune/hematologic diseases.

### Hospital course and treatment

**Figure 3:**
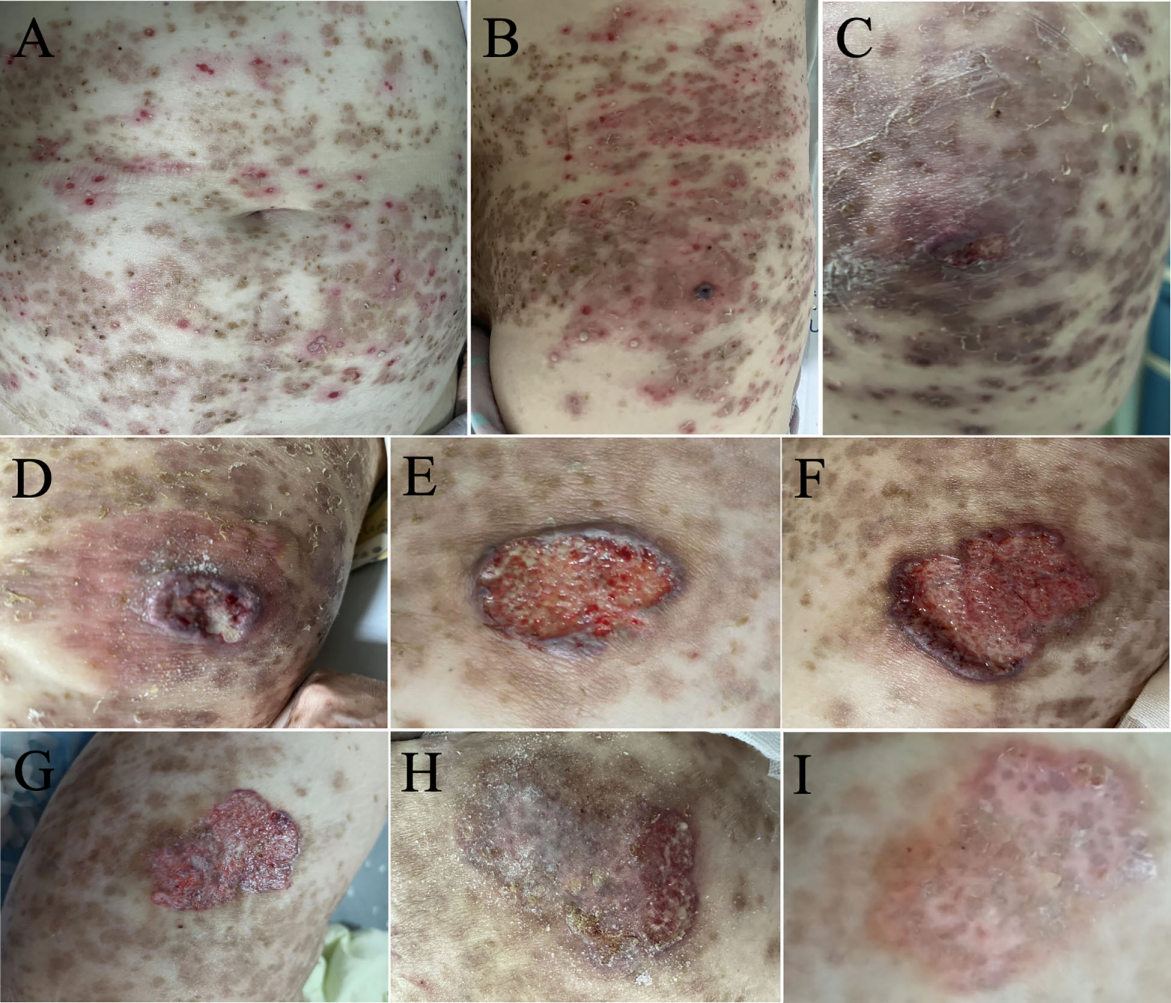
The patient’s skin rash showed significant improvement on the third day of hospitalization **(A)**. However, a new ulcer developed on the patient’s left lumbar region on the third day after admission **(B)**, which significantly enlarged one week later **(C, D)**. Successful disease control was achieved with a combination of dapsone and low-dose corticosteroids **(E, F)**. In follow-up, the new ulcer on the left waist showed significant improvement. One week after discharge, The wound was clean and superficial, with visible granulation tissue and epithelial islands at the edges. The steroid dosage was reduced to prednisone 30 mg/day orally **(G)**. Two weeks after discharge, the new ulcer had mostly healed, and the steroid was further reduced to prednisone 25 mg/day orally **(H)**. Three weeks after discharge, the new ulcer had completely healed, and the steroid dosage was maintained at prednisone 25 mg/day orally **(I)**.

The patient was continued on her pre-admission methylprednisolone (8 mg/day) and total glucosides of paeony (600 mg/day). Topical 0.05% halometasone/2% fusidic acid was applied (**Figure 3A**).

On day 3, a new ulcer on the left waist, clinically consistent with PG, appeared (**Figures 3B–D**). Given her history of osteoporosis and prior tuberculosis, treatment with cyclosporine or biologic agents was deferred. A regimen of dapsone combined with low-dose corticosteroids was initiated.

Dapsone was started at 25 mg/day and, in the absence of the HLA-B*13:01 allele and with stable haematological monitoring, was gradually increased to 100 mg/day. Concurrently, intravenous methylprednisolon (28 mg/day) was administered and later transitioned to oral prednisone (35 mg/day). Local wound care was provided.

*Outcome:* The patient responded well (**Figures 3E, F**). At follow-up (1–3 weeks post-discharge), no new lesions appeared, existing ulcers healed significantly, and corticosteroids were successfully tapered (**Figures 3G–I**). Unfortunately, the patient later self-discontinued dapsone against medical advice.

## Discussion

This case presents a rare co-occurrence of two neutrophilic dermatoses (ND)—IgA pemphigus and pyoderma gangrenosum (PG)—in association with IgA-type MGUS. ND are characterised by sterile neutrophil-rich skin infiltrates and are increasingly recognised to be associated with systemic conditions, particularly haematological disorders (1).

The patient’s 19-year diagnostic odyssey, from pustular vasculitis to the final diagnoses, underscores the clinical and histopathological overlap within the ND spectrum. The definitive diagnosis of IgA pemphigus was made on clinicopathological grounds despite negative immunofluorescence, a recognised scenario in some cases (4). The subsequent development of classic PG lesions further illustrates the polymorphic nature of these disorders.

The detection of IgA-κ MGUS is a salient feature. Associations between ND and paraproteinaemias, particularly IgA type, are documented (2, 3). Notably, in this case, initial immunofixation electrophoresis and bone marrow aspiration did not reveal MGUS. However, upon disease progression, MGUS was identified during disease progression by subsequent tests. The pathogenic link may involve paraprotein-mediated effects on neutrophil function or chemotaxis (5). Current evidence suggests that IgA immunoglobulinemia may contribute to pathogenesis by altering neutrophil function in both IgA pemphigus and PG associated with MGUS (6, 7). This association necessitates vigilant long-term monitoring for progression to overt malignancy, such as multiple myeloma. Additionally, given the patient’s history of cholecystectomy and constipation, examination of gut microbiota could provide insights into IgA-related mechanisms, potentially offering more targeted therapeutic clues. Unfortunately, the patient declined further investigations, and long-term follow-up is planned.

Several therapeutic options are available for ND. In IgA pemphigus, dapsone is often used as first-line treatment, either alone or in combination with immunosuppressants such as mycophenolate mofetil, methotrexate, or azathioprine, as well as phototherapy or acitretin (8). For refractory cases, biological agents including TNF-α inhibitors, intravenous immunoglobulin, or plasma exchange may be considered (6). In PG, thalidomide has shown efficacy by modulating TNF-α release and inhibiting neutrophil chemotaxis and phagocytosis (9, 10). Therapeutic management was complicated by comorbidities. Given the coexistence of ND and MGUS in this patient, potential treatments could include corticosteroids, dapsone, thalidomide, cyclosporine, or biologics targeting TNF-α, IL-17, or IL-36. However, thalidomide was not reintroduced due to prior resistance, and cyclosporine/biologics were deferred considering the patient’s economic constraints and history of tuberculosis (11). The history of tuberculosis and osteoporosis limited the use of potent immunosuppressants like cyclosporine and long-term high-dose corticosteroids. Dapsone, a mainstay for IgA pemphigus and often effective in PG, was chosen (12, 13). The combination with low-dose steroids induced a favourable response, highlighting a pragmatic approach in complex cases. Pre-treatment screening for glucose-6-phosphate dehydrogenase deficiency and the HLA-B*13:01 allele (associated with dapsone hypersensitivity) is crucial (14). During follow-up, skin lesions were significantly controlled. Dapsone was maintained at a target dose of 50–150 mg/day, tailored to the lowest effective dose for symptom control (8). Unfortunately, the patient later discontinued dapsone without medical advice.

In conclusion, ND, such as IgA pemphigus and pyoderma gangrenosum, could coexist and might be associated with haematological disorders like MGUS. Consider ND in the differential diagnosis of chronic, recurrent vesiculopustular or ulcerative eruptions. A definitive diagnosis often requires correlation of persistent clinical features with histopathology, as serological tests could sometimes be negative. Long-term, multidisciplinary management is essential, involving dermatology, haematology, and sometimes rheumatology, to address both skin disease and potential systemic associations. Therapeutic strategies must be individualised, balancing disease control against comorbidities and potential treatment-related risks.

## Data Availability

The datasets presented in this study can be found in online repositories. The names of the repository/repositories and accession number(s) can be found in the article/supplementary material.
